# Rolling the WSSe Bilayer into Double-Walled Nanotube for the Enhanced Photocatalytic Water-Splitting Performance

**DOI:** 10.3390/nano11030705

**Published:** 2021-03-11

**Authors:** Lin Ju, Jingzhou Qin, Liran Shi, Gui Yang, Jing Zhang, Li Sun

**Affiliations:** 1 School of Physics and Electric Engineering, Anyang Normal University, Anyang 455000, China; slr@aynu.edu.cn (L.S.); yg@aynu.edu.cn (G.Y.); zj@aynu.edu.cn (J.Z.); 2 School of Mechanical, Gardens Point Campus, Medical and Process Engineering, Queensland University of Technology, Brisbane, QLD 4001, Australia; 3 College of Chemistry and Chemical Engineering, Anyang Normal University, Anyang 455000, China; wlx@aynu.edu.cn; 4 Key Lab of Advanced Transducers and Intelligent Control System, Ministry of Education, Taiyuan University of Technology, Taiyuan 030024, China

**Keywords:** photocatalysis, water-splitting, WSSe bilayer, double-walled nanotube

## Abstract

For the emerging Janus transition metal dichalcogenides (TMD) layered water-splitting photocatalysts, stacking the monolayers to form bilayers has been predicted to be an effective way to improve their photocatalytic performances. To achieve this, the stacking pattern plays an important role. In this work, by means of the density functional theory calculations, we comprehensively estimate energetical stability, light absorption and redox capacity of Janus WSSe bilayer with different stacking patterns. Unfortunately, the Janus WSSe bilayer with the most stable configuration recover the out-of-plane symmetry, which is not in favor of the photocatalytic reactions. However, rolling the Janus WSSe bilayer into double-walled nanotube could stabilize the appropriate stacking pattern with an enhanced instinct dipole moment. Moreover, the suitable band edge positions, high visible light absorbance, outstanding solar-to-hydrogen efficiency (up to 28.48%), and superior carrier separation promise the Janus WSSe double-walled nanotube the potential for the photocatalytic water-splitting application. Our studies not only predict an ideal water-splitting photocatalyst, but also propose an effective way to improve the photocatalytic performances of Janus layered materials.

## 1. Introduction

Since the discovery of “Honda–Fujishima effect” in 1972 [1], overall water-splitting for hydrogen production with semiconductor-based photocatalysts has attracted extensive attentions [2,3,4]. The solar hydrogen generation is considered as a green technology to solve the growing energy crisis and environmental pollution problems [5,6,7,8,9]. The mechanism of photocatalytic water-splitting on semiconductor based photocatalysts could be elaborated as follows [6]. Under solar illumination, the photon absorption in a semiconductor gives rise to an electronic transition between the conduction and valence bands, which brings in the photo-excited carriers. After the interior and exterior recombination, the residual photo-generated electrons at the surface reduce protons in the water to form hydrogen gas (H^+^/H_2_), while the residual photo-generated holes oxidize water molecules to produce oxygen gas (O_2_/H_2_O). Normally, a high-performance water-splitting photocatalyst needs to fulfill three requirements, namely a high visible photons utilization efficiency, the excellent capability for carriers separate and transfer, and suitable band edge potentials for sufficient redox ability of photo-generated carriers. Unfortunately, an irreconcilable contradiction arises that, the high light utilization rate demands a adequately narrow band gap, however, the competent redox capability usually calls for a large band gap (≥1.23 eV). The new fast-developing two dimensional (2D) polar materials bring a new dawn for solving this problem.

For 2D polar photocatalysts, Yang et al. presented that, because of the existing polarization, the top of valence band and the bottom of conduction band will distribute in the two opposite sides, bringing in a potential difference, which will boost the redox capacity of photoexcited carriers, lowering the demand of band gap [10]. They predicted that, with the help of large surface potential energy difference (Δ*Φ* = 10.01 eV), the surface-functionalized boron nitride bilayers with a tiny band gap (*E*_g_ = 0.85 eV) could match redox levels of water-splitting reactions, making the photocatalysis process sensitive to infrared light [10]. According to Yang’s findings above, it is believable that, if there are some method that could increase the surface potential difference and decrease the band gap for the 2D polar photocatalysts, their redox capacity and light absorption could be enhanced at the same time. For the recently emerged Janus MoSSe layered materials, it has been theoretically predicted that, stacking the monolayers to form bilayers could effectively narrow the band gap [11,12], causing a reinforced optical absorption, which is similar to the cases of C_3_N_4_ and PtSSe [13,14]. In the meantime, the dipole moment of Janus MoSSe layered materials, which is represented by the plane electrostatic potential difference between the two surfaces, has been found to almost linearly increase with the growing thickness [11,12,15]. Furthermore, the observed type-II band alignment in the Janus MoSSe bilayers could suppress the recombination of photo-excited carriers for the thorough-going spatial separation [16]. Thereby, compared with the Janus MoSSe monolayers, the bilayer samples have a more excellent photocatalytic performance. Lately, due to the excellent optical absorption and high carrier separation, Janus WSSe monolayer has been also reported to possess a great potential for the application of photocatalytic overall water-splitting [17]. However, for the Janus WSSe bilayer, the photocatalytic properties is yet unclear.

**Figure 1:**
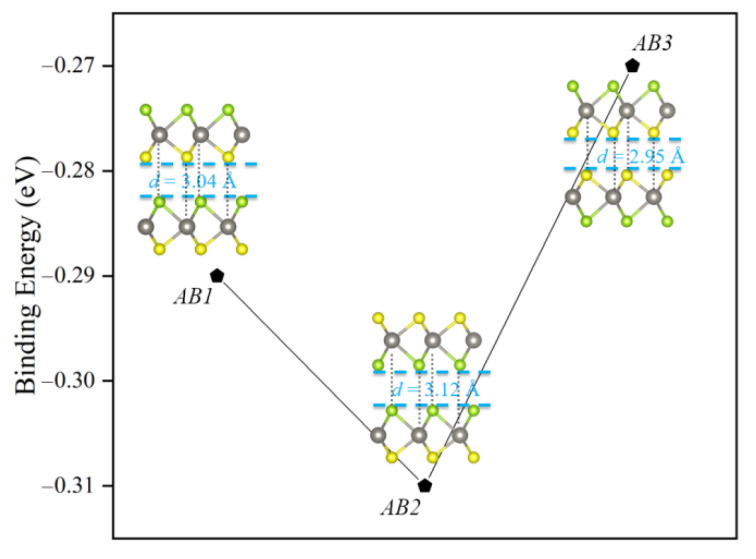
The atomic structures of WSSe bilayer with different stacking patterns (*AB1*, *AB2*, and *AB3*) and their respective binding energy.

Here, through the density functional theory (DFT) calculations, we comprehensively estimate energetical stability, light absorption and redox capacity of Janus WSSe bilayer with different stacking patterns. Unluckily, we find the Janus WSSe bilayer with the most stable configuration (*AB2* stacking style, shown in Figure 1) recovers the out-of-plane symmetry, which is to the disadvantage of the photocatalytic reactions. However, rolling the Janus WSSe bilayer into double-walled nanotube (DWNT) could stabilize the ideal stacking pattern (*AB1* stacking style, shown in Figure 1), which has a raised polarization. Moreover, the suitable band edge positions, high visible light absorbance, outstanding solar-to-hydrogen efficiency (up to 28.48%), and superior carrier separation indicate that, the Janus WSSe DWNT is potentially an high-efficiency candidate for the photocatalytic water-splitting application.

## 2. Computational Methods

In this work, we employ the Vienna Ab initio Simulation Package (VASP) (version 5.3) software to carry out the DFT calculations for both geometry relaxations and electronic structures [18,19]. We use the projector augmented wave (PAW) pseudo potentials to describe the electron–ion interaction [20,21]. We choose generalized gradient approximations of Perdew–Burke–Ernzerhof (GGA-PBE) as the exchange-correlation functional [22]. In order to avoiding the interactions with neighboring mirror images, we set a 20 Å vacuum space vertical to each Janus WSSe bilayer, and more than 15 Å vacuum spaces along *x* and *y* directions for each Janus WSSe DWNT, whose periodic boundary condition is along the *z* axis. We apply the DFT-D3 approach of Grimme to address the van der Waals (vdW) force [17,23]. The Brillouin zone is regulated with a 9 × 9 × 1 gamma-pack k-mesh for the Janus WSSe bilayers, and a 1 × 1 × 7 one for the Janus WSSe double-walled nanotube. The cutoff energy is set to 500 eV, and the convergence criteria for the force and energy is 10^−^^2^ eV/Å and 10^−5^ eV, respectively. Although tungsten is a heavy element, since the effect of spin-orbital coupling (SOC) on the band gap of WSSe monolayer has been verified to be unremarkably [17], here we do not apply the SOC correction in our calculations for saving the computing resource. More computational details of theoretical STH efficiency and DWNT surface Δ*Φ* can be found in the Supplementary Materials.

## 3. Results and Discussion

### 3.1. Geometric and Electronic Structures of Janus WSSe Bilayer

In this study, we choose the *AB* stacking mode (translation symmetry) for the Janus WSSe bilayers, which has been widely demonstrated to be more stable than the other stacking modes for transition metal dichalcogenides layered materials [11,12,15,24,25]. In the *AB* stacking mode, the S/Se (W) atom of the first layer locate above the W (S/Se) atom of the second layer. As depicted as in Figure 1, based on the atomic species at the interface, three kinds of WSSe bilayer stacking configurations are considered, namely S-Se (denoted as *AB1*), Se-Se (denoted as *AB2*), and S-S (denoted as *AB3*) with the point groups of *C*_3v_, *D*_3d_, and *D*_3d_, correspondingly. After the full optimization, the lattice constants (*a* = *b*) of *AB1*, *AB2*, and *AB3* stacking configurations nearly are the same (about 3.25 Å) (listed in Table 1), which well agrees with the result (3.228 Å) in the previous report [24]. 

In the beginning, we check the stacking-dependent stability by calculating the binding energy$\;E_{b}$, which could be obtained with the following equation [13,24]:

(1) $$E_{b}=E_{\mathrm{BL}}-2\times E_{\mathrm{ML}}$$

where $E_{\mathrm{ML}}$ and $E_{\mathrm{BL}}$ are separately the total energies of Janus WSSe monolayer and bilayer. Under this definition, a more negative$\;E_{b}$ value indicates a higher energetical stability. According to the calculated binding energy listed in the Table 1, the *AB2* stacking configuration has the most negative$\;E_{b}$ among all the three cases, forecasting the most stable stacking approach for the Janus WSSe monolayer to form its bilayer.

**Figure 2:**
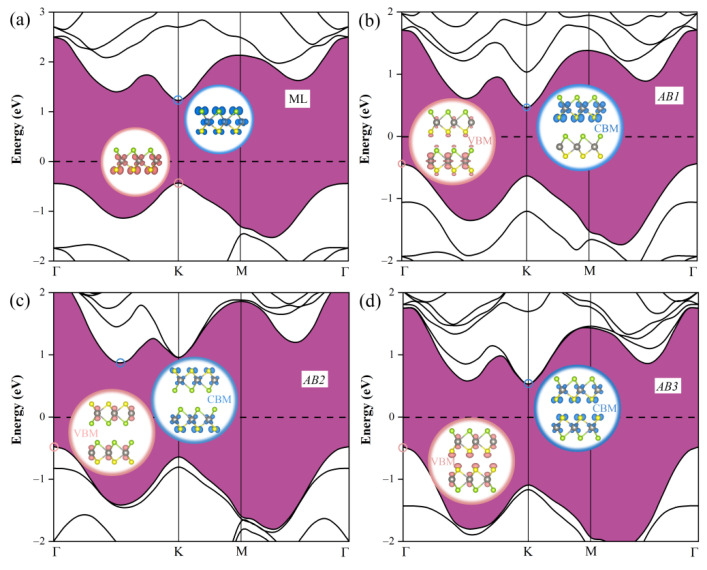
The electronic band structure of WSSe (**a**) monolayer (ML) and (**b**) *AB1*, (**c**) *AB2*, and (**d**) *AB3* bilayers. The inserts show the spatial distributions of conduction band minimum (CBM) (blue areas) and valence band maximum (VBM) (pink areas), respectively. We set the isosurface value to 0.018 e/Å^3^.

Next, we study the electronic properties of Janus WSSe bilayer by investigating their electronic band structures with various stacking models. Here, we choose the results achieved by PBE functional instead of Heyd−Scuseria−Ernzerhof (HSE06) hybrid functional or G_0_W_0_ calculations. This is because that, as shown in Figure 2a, the calculated band gap of WSSe monolayer at PBE level (1.68 eV) is closer to the experimental optical gap (1.83 eV) [26] than the one at the HSE06 level (2.13 eV) or at the G_0_W_0_ level (2.68 eV) [17]. Our treatment agrees with the one applied in the previous studies on Janus TMD studies [27,28,29]. As plotted in Figure 2, all these Janus WSSe bilayers present indirect band gap, different from the case of its monolayer. Specifically, for the Janus WSSe monolayer, the conduction band minimum (CBM) and valence band maximum (VBM) are both at the *K* point [17]; nevertheless, as to the bilayer cases, all the VBM moves to the *Γ* point, meanwhile the CBM of *AB1* and *AB3* stacking modes still stay at the *K* point, and the one of *AB2* stacking mode lies on the *Γ*-*K* path. The direct−indirect transition of band gap, caused by the forming bilayer, is able to suppress the photogenerated electron-hole recombination, hopefully raising the quantity of free carriers. Furthermore, as summarized in the Table 1, all the band gaps of bilayer (0.90, 1.36, and 1.01 eV for *AB1*, *AB2* and *AB3* modes, respectively) are obviously smaller than the one of monolayer (1.68 eV). The narrowed band gap in these bilayers is conducive to improve the light absorption efficiency, which will be discussed later. Intriguingly, the spatial distribution of CBM and VBM in the Janus WSSe bilayers depends on the stacking pattern. As illustrated in the inset of Figure 2, for the *AB1* stacking pattern, the CBM distributes at the first layer, while the VBM is dominantly distributed at the second layer. However, for the *AB2* and *AB3* stacking patterns, both VBM and CBM uniformly spread over the both layers. Under illumination, the photo-excited electron jump to the CBM, at the same time, the photo-excited hole stay at the VBM. Hence, the locational separation of CBM and VBM normally could lower the carrier recombination. Consequently, from the perspective of increasing the free photo-generated carrier number, the *AB1* stacking pattern is a better choice. It is also better than the monolayer, where both the CBM and VBM are located partially at the W atomic layer (see the inset of Figure 2a).

### 3.2. Redox Potential of the Photoexcited Carriers in Janus WSSe Bilayer

In order to overall split water, the adequate redox capacities of photo-generated carriers require that, in photocatalysts, the CBM position should be higher than the H^+^/H_2_ reduction potential (−4.44 eV at pH = 0), while the VBM one lower than the H_2_O/O_2_ oxidation potential (−5.67 eV at pH = 0). For the polar materials, such as Janus TMD and ferroelectrics, the intrinsic dipole has a strong impact on the CBM and VBM positions [17,30,31,32]. Usually, for a given 2D polar material, the intrinsic dipole can be evaluated by the Δ*Φ* [31,32]. By this means, we study the stacking-dependent dipole moment of the Janus WSSe bilayers. As illustrated in Figure S1, due to the recovery of out-of-plane symmetry, the Δ*Φ* is 0 eV in the *AB2* and *AB3* stacking configurations. However, as to the case of *AB1* stacking configuration, Δ*Φ* (1.37 eV) is nearly twice the one (0.73 eV) of Janus WSSe monolayer [17], which indicates an enhanced intrinsic dipole moment.

**Figure 3:**
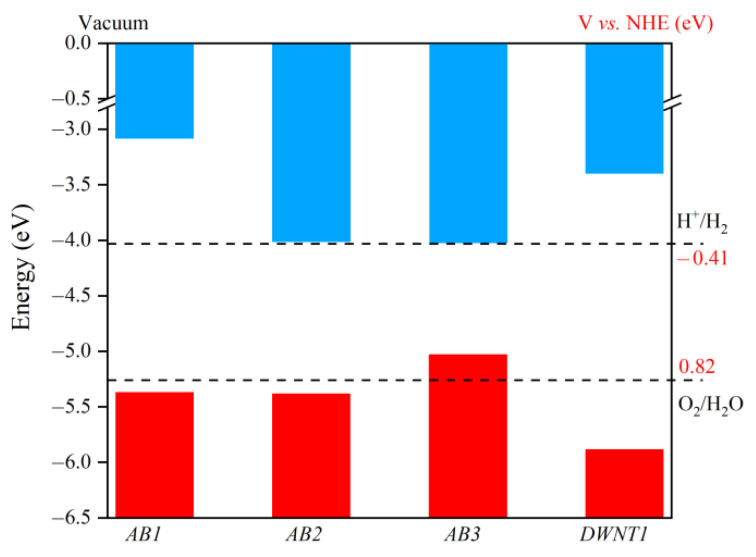
The potential of the CBM (blue region) and VBM (red region) of WSSe bilayer with different stacking patterns (*AB1*, *AB2*, and *AB3*) and WSSe *DWNT1*, with respect to the vacuum level (labeled as 0 eV). The dashed lines mark the oxidation potential of O_2_/H_2_O and reduction level of H^+^/H_2_. The pH is set to 7.

After taking the Δ*Φ* in consideration, we evaluate the redox potential of the photoexcited carriers in Janus WSSe bilayer through Yang’s method [7,10]. As shown in Figure S2, since both the oxidation potential of O_2_/H_2_O and reduction potential of H^+^/H_2_ lie in the gap, the samples with *AB1* and *AB2* stacking patterns meet the redox potentials requirements for overall water-splitting reactions at pH = 0. In order to make the practical photocatalytic applications low-cost and eco-friendly, these overall water-splitting reactions would better take place in neutral environment (pH = 7). Because the pH dependence of band edge positions is exactly consistent with that of water redox potentials (0.059 × pH) [33,34] *AB1* and *AB2* configurations in theory are capable to catalyze water-splitting at pH = 7 (see Figure 3) Whereas, for the *AB3* configuration, the VBM potential is higher than the O_2_/H_2_O oxidation potential, making it incapable for the oxygen evolution reaction (OER). For the hydrogen evolution reaction (HER), the CBM location of *AB2* and *AB3* configurations is too much close to the H^+^/H_2_ reduction level, so that their hydrogen evolution performances probably cannot be high. However, with the help of enhanced Δ*Φ*, the CBM location of *AB1* mode is greatly lifted, which is obviously higher than the H^+^/H_2_ reduction level, promising a outstanding hydrogen evolution ability.

### 3.3. Optical Absorption and STH Efficiency of Janus WSSe Bilayer

**Figure 4:**
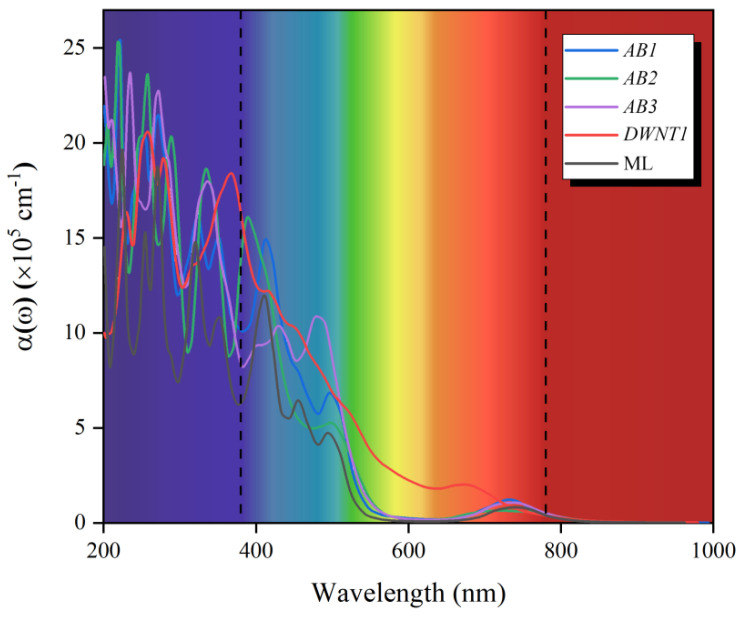
Optical absorption coefficients *α*(ω) for the WSSe ML, WSSe bilayer with different stacking patterns (*AB1*, *AB2*, and *AB3*), and WSSe *DWNT1*.

With regard to an ideal photocatalyst, the solar absorptivity, which could be assessed by the absorption coefficient a(ω), should be good. Hereon, we study the stacking-dependent a(ω) of Janus WSSe bilayer based on the formula below [32,35]:

(2) $$a\left(\omega \right)=\sqrt{2}\frac{\omega }{c}{\left(\sqrt{\varepsilon_{1}{\left(\omega \right)}^{2}+\varepsilon_{2}{\left(\omega \right)}^{2}}-\varepsilon_{1}\left(\omega \right)\right)}^{\frac{1}{2}}$$

where *ε*_1_ and *ε*_2_ separately stand for the real and imaginary part of dielectric function. As plotted in Figure 4, among the visible range (380–780 nm), these three kinds of Janus WSSe bilayers each has several significant absorption peaks (>10^5^ cm^−1^), indicating that they are hopeful visible-light-response candidates. Here, we draw the a(ω) of Janus WSSe monolayer as a contrast (black line). It could be found that, nearly all the visible light absorption peaks of the bilayers exhibit red-shift, comparing with the ones of monolayer, which could be explained by the visibly narrower band gaps as noted earlier.

Normally, for a water-splitting photocatalyst, the strong light absorption capacity in the visible-light area heralds a high STH efficiency. According to the method proposed by Yang’s group [36], we estimate the energy conversion efficiency of these Janus WSSe bilayers with the data of band alignments (see Table S1) discussed above. As shown in Table 2, because of the ultrahigh light absorption efficiency (*η*_abs_) and carrier utilization efficiency (*η*_cu_), the STH efficiency (*η*_STH_) of *AB1* mode arrives 31.22%. Even considering the instinct dipole contribution into the total energy, the corrected solar-to-hydrogen efficiency (*η’*_STH_) of *AB1* mode is still up to 19.46%, far over the standard conversion efficiency of commercial applications for hydrogen production through photocatalytic water splitting (10%) [37]. It also markedly outstrips the one of Janus WSSe monolayer (11.68%) [17] and other reported photocatalysts, i.e., AgBiP_2_Se_6_ (10.04%) [30], and most M_2_X_3_ (M = Al, Ga, In; X = S, Se, Te) monolayers [36]. Nevertheless, the *η*_STH_ of *AB2* and *AB3* configurations are not as high as the one of *AB1* configuration, due to the badly low *η*_cu_. As stated before, in the *AB1* mode, the large Δ*Φ* raises the redox potentials, causing a high *η*_cu_. Whereas, the out-of-plane symmetry recovery makes the Δ*Φ* disappear in *AB2* and *AB3* configurations. Their redox potentials are therefore much lower than the ones in *AB1* configuration, so are their *η*_cu_. To sum up, based on the staking-dependent STH efficiency of Janus WSSe bilayers considered in our study, the *AB1* configuration is most suitable for the photocatalytic water-splitting application.

### 3.4. Geometric and Electronic Structures, and Photocatalytic Properties of Janus WSSe Double-Walled Nanotube

**Figure 5:**
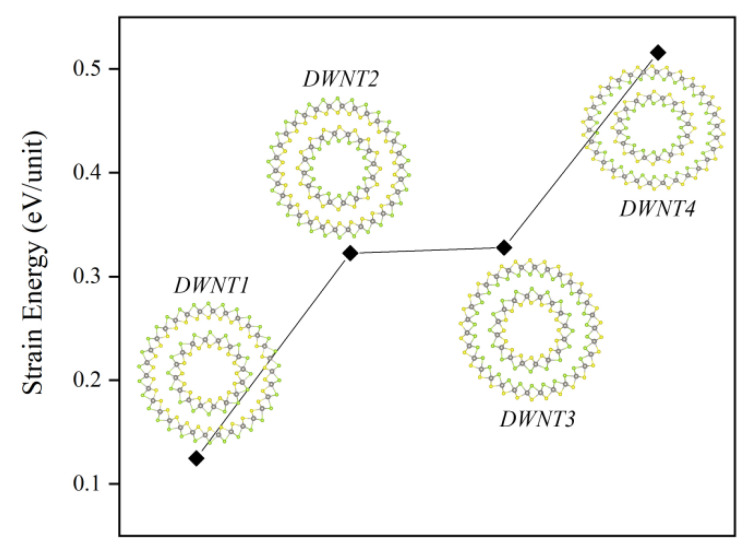
The atomic structures of WSSe double-wall nanotubes with different stacking patterns (*DWNT1*, *DWNT2*, *DWNT3*, and *DWNT4*) and their respective strain energy.

As the previous lines already suggested, due to the most negative bonding energy, the *AB2* configuration is the most stable stacking pattern among these three cases, however, its photocatalytic performance is far less excellent than the one of *AB1* mode. Therefore, making the stacking pattern of WSSe bilayer uniformly follow the *AB1* style is a hopeful way to improve its photocatalytic performance. Rolling the WSSe bilayer into double-walled nanotube may realize this. It has been reported that, for a given diameter, the Janus MoSSe nanotubes with outer shell of selenium atoms and inner shell of sulfur atoms (Se-W-S, outside→inside) have a lower strain energy than the corresponding ones with the opposite structures (S-W-Se), which can be explained that, the selenium atom has a larger radius than the sulfur atom, and the inner atoms with larger radius likely have stronger repulsive force [28]. Therefore, it can be expected that, for the Janus WSSe DWNT, the stacking pattern predictably follows the *AB1* style, which could be checked through comparing the strain energy of Janus WSSe DWNTs with different stacking patterns [38,39,40]. Since the armchair Janus MoSSe nanotube has been reported to be more energetically stable than the zigzag one [27,28], here we choose (15, 15) and (8, 8) Janus WSSe armchair nanotubes to build the Janus WSSe DWNT, where the distance between the inner and outer layers is close to the one of Janus WSSe bilayer (about 3 Å, see Figure 1). As shown in the Figure 5, four stacking configurations for the Janus WSSe DWNT are considered, namely Se-W-S-Se-W-S, Se-W-S-S-W-Se, S-W-Se-Se-W-S, and S-W-Se-S-W-Se (outer layer→inner layer), which are separately labelled as *DWNT1*, *DWNT2*, *DWNT3*, and *DWNT4*. In this work, the strain energy *E*_str_ is defined as follows:

(3) $$E_{\mathrm{str}}=\frac{E_{\mathrm{DWNT}}}{N_{\mathrm{DWNT}}}-\frac{E_{M}}{N_{M}}$$

where $E_{\mathrm{NT}}$ and$\;N_{\mathrm{NT}}\;$are the total energy and the number of unit cells in the Janus WSSe DWNTs, meanwhile,$\;E_{M}\;$and$\;N_{M}\;$are corresponding value for the WSSe monolayer, respectively. As displayed in Figure 1, the *DWNT1* configuration has the lowest strain energy among these four cases, which is in line with our expectation.

**Figure 6:**
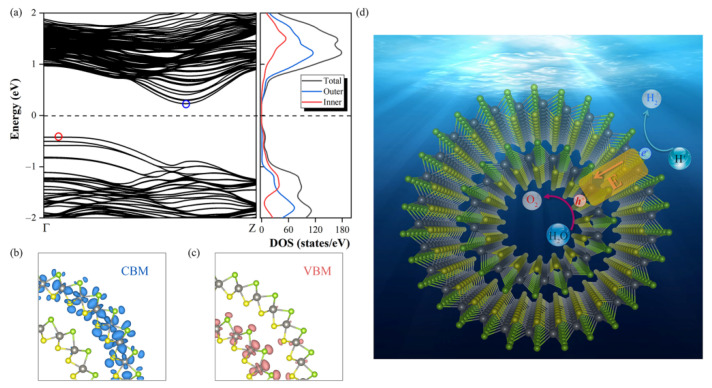
The electronic properties of WSSe *DWNT1* (**a**), including the projected density of state (right) and band structure (left). The Fermi level (labeled with the dashed line) is set to 0 eV. The spatial distributions of (**b**) CBM (blue areas) and (**c**) VBM (pink areas) of WSSe *DWNT1*. We set the isosurface value to 1 × 10^−3^ e/Å^3^. (**d**) The work mechanism of WSSe *DWNT1* for photocatalytic water-splitting.

Then we explore the electronic properties of Janus WSSe *DWNT1* by investigating its electronic band structure and density of state (DOS). As shown in Figure 6a, it exhibits an indirect band gap of 0.65 eV, which is even smaller than the one of WSSe bilayer, ensuring the intensive light absorption in the visible areas (see Figure 4, red line). Moreover, as illustrated in Figure 6b–c, similar to the case of *AB1* mode, in the Janus WSSe *DWNT1*, the CBM mainly scatter on the outer layer, while the VBM generally is located at the inner layer, which is coincident with the results of DOS (see Figure 6a). To get the Δ*Φ* between the inner and outer layers of Janus WSSe *DWNT1*, we calculate the Δ*Φ* of its component parts ((8, 8) and (15, 15) nanotubes) with corresponding building block models first. Then, based on the fitting line of the relationship between the Δ*Φ* for Janus MXY (M = Mo, W; X, Y = S, Se, Te) monolayers and the ones for their own bilayers, we estimate the Δ*Φ* of Janus WSSe *DWNT1*. More calculation details could be found in the Supplementary Materials (see Figures S3–S5). Notably, the Δ*Φ* (1.82 eV) of Janus WSSe *DWNT1* is larger than the one (1.37 eV) of *AB1* mode, indicating that rolling into DWNT could strengthen the Δ*Φ* of WSSe bilayer, which may be related to the strains appeared during the coiling processes [17]. The Δ*Φ* in the Janus WSSe *DWNT1* will generate a built-in electric field pointing from the outer shell to the inner one. As depicted in the Figure 6d, this built-in electric field effectively pushes the photoexcited electrons to run to the outer layer, meanwhile it also forces the photoexcited holes to stay at the inner layer. Besides, as illustrated in Figure 3, the band edge positions of *DWNT1* broadly straddle the standard water-splitting redox potential, which separately ensures the competent reduction ability for the HER at the outer surface, and the sufficient oxidation capacity the OER at the inner surface. What’s more, the *η’*_STH_ of *DWNT1* reach up to 28.48%, which greatly transcends the ones of corresponding bilayers. Additionally, as a complement, we also perform a computational study on the energy conversion efficiency of WSSe (15, 15) single-walled nanotube (SWNT), which is summarized in Table 2. Due to the narrower band gap (1.56 eV) and larger Δ*Φ*, the WSSe (15, 15) SWNT has an obviously higher *η’*_STH_ (22.18%) than its monolayer (*η’*_STH_ = 11.68%) [17]. This is consistent with the situation of DWNT. However, the *η’*_STH_ of the SWNT is still inferior to the one of DWNT, indicating that, similar to the case of bilayer, the photocatalytic performance of SWNT could be improved by forming DWNT as well.

## 4. Conclusions

From the geometric, electronic, optical and chemical properties, we have presented comprehensive DFT calculations to investigate stacking-dependent photocatalytic performance of the Janus WSSe bilayer. Though the *AB2* stacking sample is energetically most stable, due to the recovery of the out-of-plane symmetry, its redox capacity is fairly low, which seriously restricts the STH conversion efficiency. Moreover, the spatial overlap of CBM and VBM probably causes a high carrier recombination. Fortunately, changing the stacking pattern into *AB1* style could effectively overcome these drawbacks above. Based on the strain energy, we find that rolling the Janus WSSe bilayer into double-walled nanotube is a promising path to stably realize the *AB1* configuration. Notably, because of the enhanced intrinsic dipole, the STH conversion efficiency of the WSSe *DWNT1* is even higher than that of the *AB1* configuration. Therefore, constructing the double-walled nanotubes is a promising approach to improve the photocatalytic performances for the Janus WSSe layered materials.

## Figures and Tables

**Table 1:** Detailed information of Janus WSSe bilayers with different stacking patterns. Calculated lattice constants (*a* and *b*), binding energy (*E*_b_), surface potential energy difference (Δ*Φ*), and band gap (*E*_g_).

Stacking Patterns	*a* = *b* (Å)	*E*_b_ (eV)	Δ*Φ* (eV)	*E*_g_ (eV)
*AB1*	3.253	−0.29	1.37	0.90
*AB2*	3.251	−0.31	0	1.36
*AB3*	3.254	−0.27	0	1.01

**Table 2:** For Janus WSSe bilayers with different stacking patterns and Janus WSSe nanotubes (*DWNT1* and (15, 15) SWNT), the Light Absorption Efficiency *η*_abs_, Carrier Utilization Efficiency *η*_cu_, STH Conversion Efficiency *η*_STH_, and Corrected STH Conversion Efficiency *η*’_STH_.

Configuration	*η_abs_* (%)	*η*_cu_ (%)	*η*_STH_ (%)	*η’*_STH_ (%)
*AB1*	81.11	38.50	31.22	19.46
*AB2*	55.59	20.38	11.33	--
*AB3*	54.69	10.01	5.47	--
*DWNT1*	76.38	64.44	49.22	28.48
(15, 15)	57.80	47.86	27.66	22.18

## Data Availability

The data presented in this study are available in Supplementary Materials.

## Supplementary Materials

The following are available online at https://www.mdpi.com/2079-4991/11/3/705/s1, Figure S1: Planar average electrostatic potential energy of the bilayer WSSe with (a) *AB1*, (b) *AB2*, and (c) *AB3* stacking models, respectively, Figure S2: The potential of the CBM (blue region) and VBM (red region) of WSSe bilayer with different stacking patterns (*AB1*, *AB2*, and *AB3*) and WSSe *DWNT1*, with respect to the vacuum level (labeled as 0 eV), Figure S3: Average electrostatic potentials in the plane normal to the tube axis for the whole structure of Janus WSSe *DWNT1*, Figure S4: The fitting line of the relationship between the electrostatic potential difference for Janus MXY (M = Mo, W; X, Y = S, Se, Te) monolayers (Δ*Φ*_monolayer_) and the ones for their own bilayers (Δ*Φ*_bilayer_), Figure S5: Average electrostatic potentials in the plane normal to the tube axis for the whole structure (left) and building block (right) of (a) (8, 8) and (b) (15, 15) Janus WSSe armchair nanotubes, Table S1: Over-Potential for Hydrogen Evolution Reaction *χ*(H_2_), Over-Potential for Oxygen Evolution Reaction *χ*(O_2_), Direct Band Gaps, and Difference of Electrostatic Potential (Δ*Φ*) of Janus WSSe A-NTs.
