# Supplementary material for: Rolling the WSSe Bilayer into Double-Walled Nanotube for the Enhanced Photocatalytic Water-Splitting Performance
Source: Nanomaterials (Basel). 2021 Mar 11;11(3):705. doi: 10.3390/nano11030705 (PMC8000809; doi:10.3390/nano11030705)
Supplement: Supplementary file 1 [file nanomaterials-11-00705-s001.pdf]

nanomaterials

Supplementary Materials

Rolling the WSSe Bilayer into Double-Walled Nanotube for the Enhanced Photocatalytic Water-Splitting Performance

Lin Ju [1,2,*], Jingzhou Qin [3], Liran Shi [1], Gui Yang [1], Jing Zhang [1] and Li Sun [4,*]

[1] School of Physics and Electric Engineering, Anyang Normal University, Anyang, 455000, China; slr@aynu.edu.cn (L.S.); yg@aynu.edu.cn (G.Y.); zj@aynu.edu.cn (J.Z.)
[2] School of Mechanical, Medical and Process Engineering, Queensland University of Technology, Gardens Point Campus, QLD 4001, Brisbane, Australia
[3] College of Chemistry and Chemical Engineering, Anyang Normal University, Anyang 455000, China; wlx@aynu.edu.cn
[4] Key Lab of Advanced Transducers and Intelligent Control System, Ministry of Education, Taiyuan University of Technology, Taiyuan, 030024, China
* Correspondence: sdu_JL@163.com or julin@aynu.edu.cn (L.J.); sunlitut@163.com or sunli02@tyut.edu.cn (L.S.)

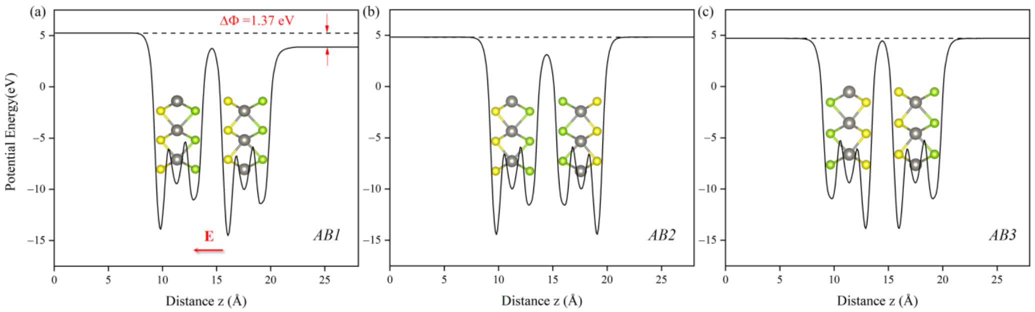

Figure S1. Planar average electrostatic potential energy of the bilayer WSSe with (**a**) *AB1*, (**b**) *AB2*, and (**c**) *AB3* stacking models, respectively. $\Delta\Phi$ is the potential energy difference.

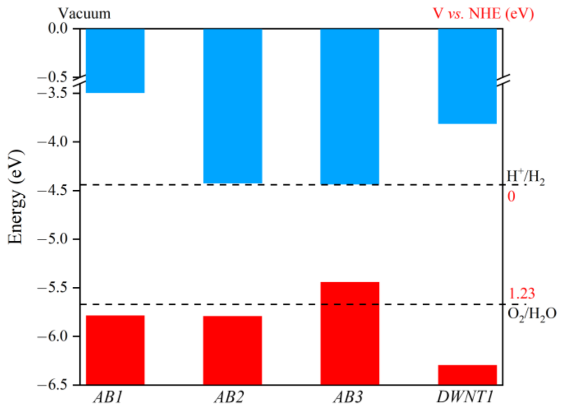

Figure S2. The potential of the CBM (blue region) and VBM (red region) of WSSe bilayer with different stacking patterns (*AB1*, *AB2*, and *AB3*) and WSSe *DWNT1*, with respect to the vacuum level (labeled as 0 eV). The dashed lines mark the oxidation potential of O_2/H_2O and reduction level of H^+/H_2. The pH is set to 0.

The potential energy difference (Δ*Φ*) between the inner shell and outer shell of Janus WSSe *DWNT1*

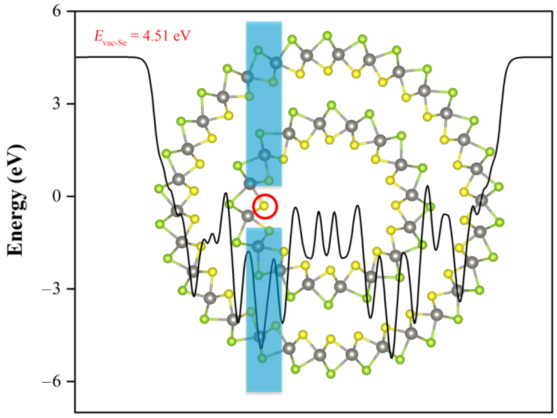

Figure S3. Average electrostatic potentials in the plane normal to the tube axis for the whole structure of Janus WSSe *DWNT1*.

For the *DWNT1* configuration, the electrostatic potential of the inside layer (as labelled with a red circle in Figure S3) could not be obtained directly with a whole structure, because in the plane normal to the tube axis, the electrostatic potential of the neighboring atoms (in the blue region in Figure S3) will be a distracter, which is hardly eliminated from the output file.

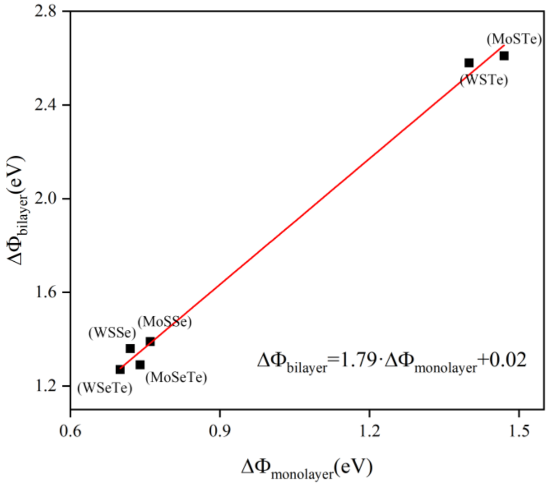

Figure S4. The fitting line of the relationship between the electrostatic potential difference for Janus MXY (M = Mo, W; X, Y = S, Se, Te) monolayers ($\Delta\Phi_{monolayer}$) and the ones for their own bilayers ($\Delta\Phi_{bilayer}$). All the data are from the previous literature [1].

It has been reported that, as plotted in Figure S4, the potential difference of Janus MXY (M = Mo, W; X, Y = S, Se, Te) bilayers ($\Delta\Phi_{bilayer}$) with *AB1* stacking pattern are almost double that ($\Delta\Phi_{monolayer}$) of the MXY monolayer [1]. Based on the fitting line, the relationship between them can be expressed as follows,

$$\Delta\Phi_{bilayer} = 1.79 \times \Delta\Phi_{monolayer} + 0.02 \quad \text{(S1)}$$

Hereby, our idea of obtaining the Δ*Φ* of *DWNT1* is that, we separately calculated the Δ*Φ* of its component parts ((8, 8) and (15, 15) nanotubes) first; then, based on the Equation

S1, we estimate the $\Delta\Phi$ of Janus WSSe *DWNT1*. Similar to the case of *DWNT1*, the $\Delta\Phi$ of Janus WSSe single-walled nanotube could not be gained directly with the whole structure either. Here we innovatively separate the building block (in the blue dotted box in Figure S5) from the whole structure, and compute its average electrostatic potentials to obtain $\Delta\Phi$ for each Janus WSSe armchair single-walled nanotube. As shown in Figure S5, the $\Delta\Phi$ of (8, 8) and (15, 15) Janus WSSe armchair nanotubes is 1.08 eV and 0.93 eV. Then we put the average of them (1.01 eV) into the Equation S1, so that the $\Delta\Phi$ of Janus WSSe *DWNT1* is 1.82 eV.

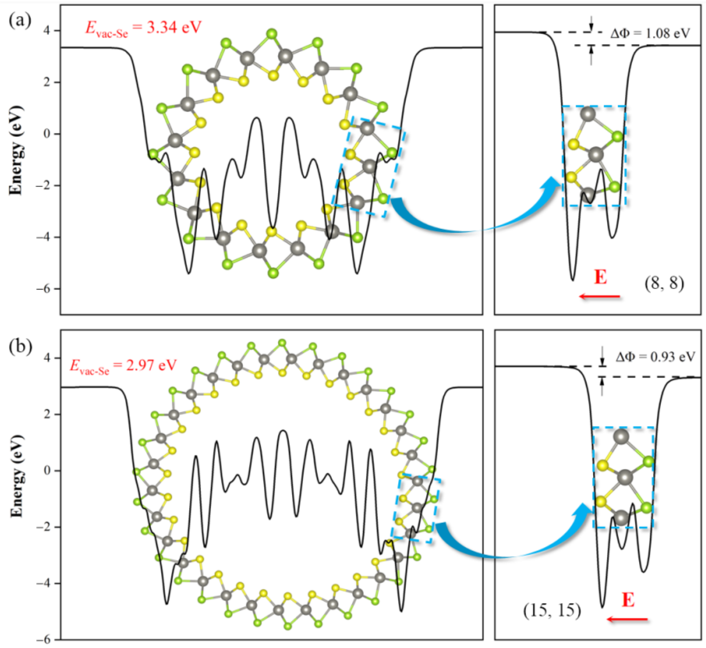

Figure S5. Average electrostatic potentials in the plane normal to the tube axis for the whole structure (left) and building block (right) of (a) (8, 8) and (b) (15, 15) Janus WSSe armchair nanotubes.

The solar-to-hydrogen (STH) efficiency

The STH efficiency is evaluated using the methods proposed by Yang et al. [2]. According to the reaction process, STH efficiency is defined as the product of the efficiency of light absorption (η_{abs}) and carrier utilization (η_{cu}).

$$\eta_{\mathrm{STH}} = \eta_{\mathrm{abs}} \times \eta_{\mathrm{cu}} \qquad (S2)$$

The efficiency of light absorption is defined as:

$$\eta_{\mathrm{abs}} = \frac{\int_{E_{\mathrm{g}}^{\mathrm{d}}}^{\infty} P(h\omega)d(h\omega)}{\int_{0}^{\infty} P(h\omega)d(h\omega)} \qquad (S3)$$

where $P(h\omega)$are the AM1.5G solar energy flux at the photon energy $h\omega$, and $E_{\mathrm{g}}^{\mathrm{d}}$ is the direct band gap of the photocatalyst, according the previous literatures [2,3]. The denominator represents the total power density of the reference sunlight spectrum (AM1.5G) and the numerator gives the light power density absorbed by the photocatalyst.

The efficiency of carrier utilization (η_{cu}) is defined as:

$$\eta_{\mathrm{cu}} = \frac{\Delta G_{\mathrm{H_2O}} \int_{E}^{\infty} \frac{P(h\omega)}{h\omega} d(h\omega)}{\int_{E_{\mathrm{g}}^{\mathrm{d}}}^{\infty} P(h\omega)d(h\omega)} \qquad (S4)$$

where $\Delta G_{\mathrm{H_2O}}$is the free energy of water splitting (1.23 eV) and the rest of numerator represents the effective photocurrent density. Here, E represents the photon energy that can be actually utilized in the process of water splitting.

$$\mathrm{E} = \begin{cases} E_{\mathrm{g}}^{\mathrm{d}}, (\chi(\mathrm{H_2}) \geq 0.2, \chi(\mathrm{O_2}) \geq 0.6) \\ E_{\mathrm{g}}^{\mathrm{d}} + 0.2 - \chi(\mathrm{H_2}), (\chi(\mathrm{H_2}) < 0.2, \chi(\mathrm{O_2}) \geq 0.6) \\ E_{g}^{d} + 0.6 - \chi(\mathrm{O_2}), (\chi(\mathrm{H_2}) \geq 0.2, \chi(\mathrm{O_2}) < 0.6) \\ E_{\mathrm{g}}^{\mathrm{d}} + 0.8 - \chi(\mathrm{H_2}) - \chi(\mathrm{O_2}), (\chi(\mathrm{H_2}) < 0.2, \chi(\mathrm{O_2}) < 0.6) \end{cases} \qquad (S5)$$

The intrinsic electric filed does positive work for the electron–hole separation during the process of photocatalytic water splitting. Therefore, this part of work should be added into the total energy, and then the corrected STH efficiency of photocatalytic water splitting with vertical intrinsic electric filed is calculated as:

$$\eta'_{\mathrm{STH}} = \eta_{\mathrm{STH}} \times \frac{\int_{0}^{\infty} P(h\omega)d(h\omega)}{\int_{0}^{\infty} P(h\omega)d(h\omega) + \Delta\Phi \int_{E_{\mathrm{g}}^{\mathrm{d}}}^{\infty} \frac{P(h\omega)}{h\omega} d(h\omega)} \qquad (S6)$$

where $\Delta\Phi$ is the vacuum level difference on the two respective surfaces. The $\chi(\mathrm{H_2})$, $\chi(\mathrm{O_2})$, E_g^d, and $\Delta\Phi$ for all samples considered in our study are listed in the Table S1.

Table S1. Over-Potential for Hydrogen Evolution Reaction $\chi(H_2)$, Over-Potential for Oxygen Evolution Reaction $\chi(O_2)$, Direct Band Gaps, and Difference of Electrostatic Potential ($\Delta\Phi$) of Janus WSSe A-NTs.

Configuration	$\chi(H_2)$ (eV)	$\chi(O_2)$ (eV)	E_g^d (eV)	$\Delta\Phi$ (eV)
AB1	0.94	0.11	1.10	1.37
AB2	0.01	0.12	1.60	0.00
AB3	0.00	−0.23	1.62	0.00
DWNT1	0.62	0.62	1.19	1.82
(15, 15)	0.79	0.47	1.56	0.93

References

1. Xia, C.; Xiong, W.; Du, J.; Wang, T.; Peng, Y.; Li, J. Universality of electronic characteristics and photocatalyst applications in the two-dimensional Janus transition metal dichalcogenides. *Phys. Rev. B* **2018**, *98*, 165424.
2. Fu, C. F.; Sun, J.; Luo, Q.; Li, X.; Hu, W.; Yang, J., Intrinsic Electric Fields in Two-dimensional Materials Boost the Solar-to-Hydrogen Efficiency for Photocatalytic Water Splitting. *Nano Lett.* **2018**, *18*, 6312–6317.
3. Yang, H.; Ma, Y.; Zhang, S.; Jin, H.; Huang, B.; Dai, Y., GeSe@SnS: Stacked Janus structures for overall water splitting. *J. Mater. Chem. A* **2019**, *7*, 12060–12067.
